# Variability in phytoplankton biomass and effects of sea surface temperature based on satellite data from the Yellow Sea, China

**DOI:** 10.1371/journal.pone.0220058

**Published:** 2019-08-06

**Authors:** Chunli Liu, Qiwei Sun, Qianguo Xing, Sufen Wang, Danling Tang, Donghe Zhu, Xiang Xing

**Affiliations:** 1 Marine College, Shandong University (Weihai), Weihai, China; 2 State Key Laboratory of Tropical Oceanography, South China Sea Institute of Oceanology, Chinese Academy of Sciences, Guangzhou, China; 3 College of Earth and Planetary Sciences, University of Chinese Academy of Sciences, Beijing, China; 4 Yantai Institute of Coastal Zone Research, Chinese Academy of Sciences, Yantai, China; 5 State Key Laboratory of Tropical Oceanography, Guangdong Key Laboratory of Remote Sensing, South China Sea Institute of Oceanology, Chinese Academy of Sciences, Guangzhou, China; 6 Ocean College, Zhejiang University, Zhoushan, China; Guangdong Ocean University, CHINA

## Abstract

A time series of satellite data on Chlorophyll-*a* concentration (Chl-*a*) that used ocean color was studied to determine mechanisms of phytoplankton variation in recent decade in the Yellow Sea, China during 2003–2015. The variability patterns on seasonal and inter-annual oscillation periods were confirmed using the Empirical Orthogonal Function (EOF), and Morlet wavelet transform analyses, respectively. The first EOF mode for Chl-*a* was dominated by obvious spring and fall blooms in a spatial pattern that was related to the strong mixing of the water masses from the Yellow Sea Cold Warm Mass (YSCWM) and the Yellow Sea Warm Current (YSWC) in winter. The second EOF mode for Chl-*a* showed an opposite spatial pattern between the northern and southern regions. The temporal coefficient showed differences in the timing of blooms. On an inter-annual scale, Chl-*a* indicated variation at periods of 2–4 years during 2003–2015. Chl-*a* showed a significantly negative correlation with the sea surface temperature (r = -0.21, p<0.01), with time lags of 4 months (Chl-*a* ahead). Chl-*a* was coupled with El Niño Southern Oscillation (ENSO) events, with a positive correlation (r = 0.46, p<0.01) at a lag of 3–5 months (ENSO ahead). The present study demonstrated that the variation in phytoplankton biomass was controlled primarily by water mass seasonally, and it was influenced by ENSO events on an inter-annual scale.

## Introduction

Marine phytoplankton is responsible for almost half of the primary productivity in the world [1]. What controls their abundance is a key issue for understanding the ocean’s biogeochemical cycle. Recent research has shown that global phytoplankton biomass has decreased since the past century, probably due to increased sea surface temperatures [2]. Phytoplankton biomass is affected by different factors at the regional scale [3–4]. Chlorophyll-*a* concentration (Chl-*a*) is the crucial biological parameter for simulating marine phytoplankton biomass [5]. Many driving factors (e.g., monsoons, upwelling, coastal currents, vertical mixing, wind speed, etc.) have also been identified that affect Chl-*a* at a regional scale. Sea surface temperature (SST), which reflects the thermal stratification of the ocean, was related closely to variations in phytoplankton biomass on a seasonal scale [6]. El Niño activity is characterized by anomalous warming of the SST in the tropical Pacific, which is linked to a perturbation of atmospheric circulation patterns known as the Southern Oscillation. This ocean-atmosphere coupling is called the El Niño Southern Oscillation (ENSO) [7]. ENSO is the dominant source of inter-annual climate variability in the tropical Pacific Ocean [8]. Fluctuations in phytoplankton biomass have been linked to inter-annual ENSO variability [9–14]. For instance, Hou et al. [15] found phytoplankton biomass positively correlated to the ENSO events in the Western Pacific Ocean. While, Zhao and Tang [16] found the phytoplankton biomass decreased during 1997–98 and 2002–03 EI Niño events.

**Fig 1:**
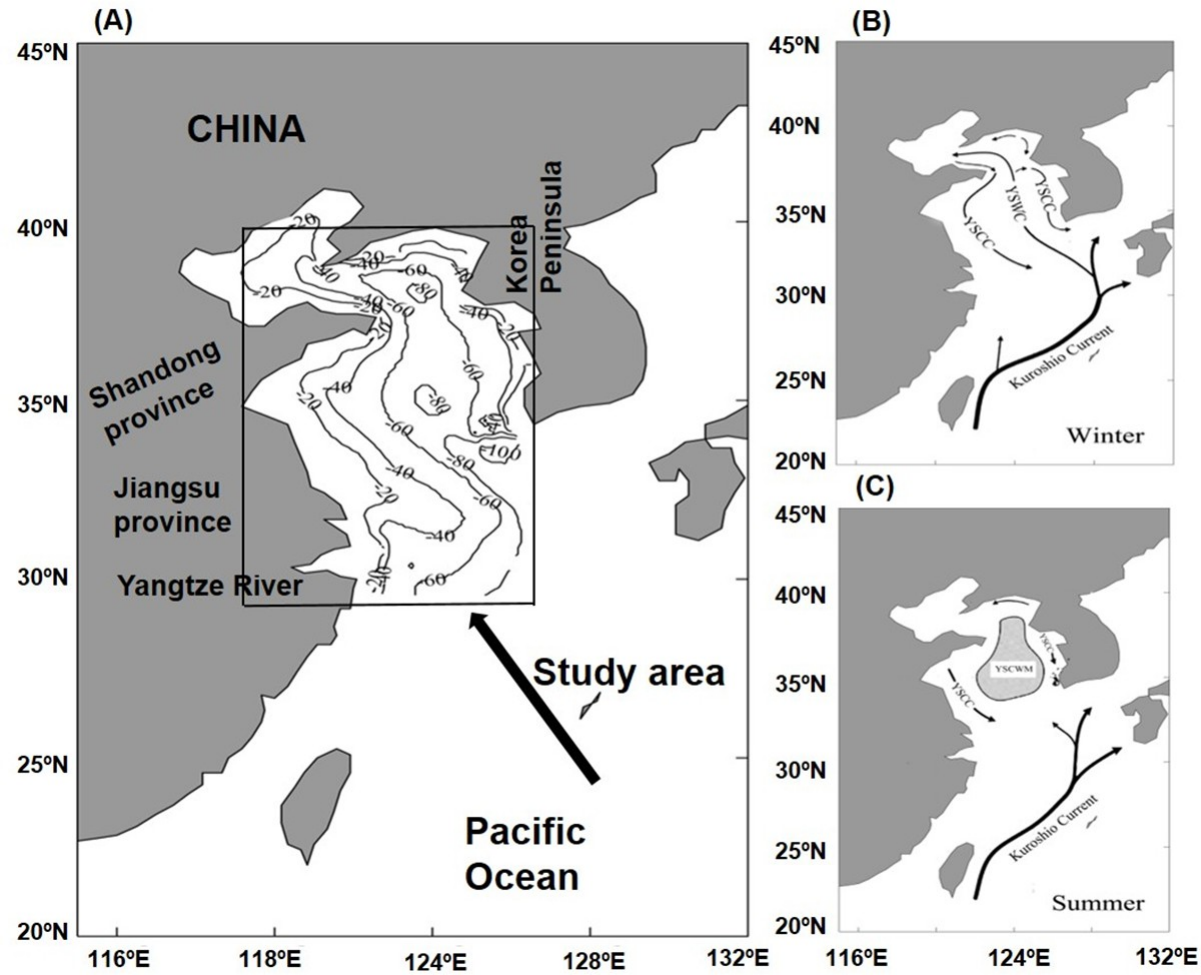
The study area in the Yellow Sea, China. (A) Location of the study area in the Yellow Sea. The isobaths are in meters (same in the below figures). (B) Major water currents during winter in the Yellow Sea. (C) Major water currents during summer in the Yellow Sea. Yellow Sea Coastal Current (YSCC), Yellow Sea Warm Current (YSWC), Yellow Sea Cold Water Mass (YSCWM).

The Yellow Sea is a relatively shallow sea and has a mean water depth of only 44 m (Fig 1A). Consequently, the Yellow Sea responds relatively quickly to climate changes [17]; in turn, it influences variability in local climate due to the air-sea feedback process. The Yellow Sea Warm Current (YSWC) flows from the southeast to the north in winter (Fig 1B) [18], and the Yellow Sea Cold Water Mass (YSCWM) (122–125° E, 33–37° N) is entrenched close to the seabed in summer (Fig 1C) [19]. These features represent the two important physical oceanographic phenomena in the Yellow Sea. Additionally, southward coastal currents flow along the coastal sides of the Yellow Sea in winter, which corresponds to the northward YSWC in the central region [20–21]. The seasonal variation in oceanic phytoplankton biomass has been recognized to change with these water circulations in the Yellow Sea, especially in the YSCWM area [22].

In recent years, researchers have reported the spatio-temporal variability in Chl-*a* over decades using satellite data [2–3, 23]. The variability in Chl-*a* was analyzed not only for the global ocean [24], but it was investigated also in the regional areas using satellite datasets [25–28]. These studies illustrated that climate-driven SST generally showed a negative correlation with Chl-*a*, partly because the increasing SST reduced the nutrients supply from the deep layer [29]. However, the detailed analyses were lacking in the Yellow Sea.

Compared with studies of seasonal changes in phytoplankton biomass in the Yellow Sea, there have been many fewer studies of inter-annual variability in phytoplankton biomass. Three notable exceptions for the Yellow Sea were the studies of Shi and Wang [30] on the seasonal and inter-annual Chl-*a* variability during 2002–2009, of Liu and Wang [31] on the Chl-*a* seasonal trend during 1997–2011, and of Yamaguchi et al. [32] that revealed seasonal and spring inter-annual variations in Chl-*a* during 1998–2007. These above studies focused on seasonal and inter-annual scales on a short period. In this study, combined the updated satellite datasets, we examined the variation in Chl-*a* seasonality during 2003–2015 in the Yellow Sea (30–40° N, 118–126° E), using the empirical orthogonal function (EOF). Also, the inter-annual temporal correlations between Chl-*a* and SST, and Chl-*a* and multivariate ENSO index (Niño 3.4) were explored using wavelet coherency analysis.

## Datasets and methodology

### Datasets

The monthly MODIS-Aqua datasets for Chl-*a* and SST from 2003–2015 were used in this study. The Chl-*a* and SST datasets were both level 3 fields at 4 km spatial resolution downloaded on the NASA ocean color webpage (http://oceancolor.gsfc.nasa.gov). The standard Chl-*a* product was derived using the OC3Mv5 algorithm, and the daytime SST 11 μm product used the 11 and 12 μm bands. The study area spanned 30–40° N in latitude and 118–126° E in longitude for the Yellow Sea region. The Niño 3.4 was acquired from NOAA's Earth System Research Laboratory (http://www.esrl.noaa.gov/psd/enso/).

### Methodology

#### Empirical Orthogonal Function (EOF)

Because the EOF and wavelet analyses generally require a complete time series of input maps without data voids, the decompositions from Data Interpolating Empirical Orthogonal Functions (DINEOF) were used to obtain complete Chl-*a* and SST datasets [33–34]. DINEOF has been used widely to construct the ocean parameters widely [25, 35–37]. EOF analysis was applied to the monthly Chl-*a* dataset. The dataset was organized in an (*M*×*N*) matrix, where M and N represented the spatial and temporal elements, respectively. Prior to EOF analysis, the temporal means in each pixel were removed for Chl-*a* from the original dataset using: $I^{\prime }(x,t)=I(x,t)-1/N\sum_{j=1}^{N}I(x,t_{j})$, where *I*′(*x,t*) are the resulting residuals (anomalies). Alternatively, the spatial means of each pixel were removed using: $I^{\prime }(x,t)=I(x,t)-1/M\sum_{j=1}^{M}I(x,t_{j}).$ For Chl-*a*, the matrix *I*(*x,t*) can be written by $I(x,t)=\sum_{n=1}^{N}an(t)Fn(x)$, where *an*(*t*) are the temporal evolution functions and *Fn*(*x*) are the spatial eigen-functions for each mode.

To assess the significance of the EOF modes, we followed the methods described by North et al. [38]. The error produced in a given EOF (*e*_*j*_) was calculated as: *e_j_* = *λ_j_* (2/*n*)^0.5^, where λ is the eigenvalue of that EOF and n is the degrees of freedom. When the difference between neighboring eigenvalues satisfied *λ_j_*−*λ*_*j*+1_≥*e_j_*, then the EOF modes represented by the former eigenvalue were significant statistically. In this study, the first two modes of Chl-*a* passed the significance test. Thus, the first two modes were used to analyze the major variability in this study.

#### Continuous wavelet transform (CWT)

The classical Fourier transform is able to localize the signal only in the frequency domain with no localization in time, but the continuous wavelet transform (CWT) can localize the signal in domains both of time and frequency domains [39]. Therefore, in this study, we used the CWT to determine the oscillation periods of DINEOF Chl-*a* on inter-annual scales. Before the CWT analysis, the seasonal mean calculated from the original dataset was removed from each pixel. The non-stationary time series at different spatial or temporal scales was decomposed into time-frequency space by the CWT, which analyzed localized variations in power and translated the mother wavelet. We used the “Morlet” wavelet as the mother wavelet, which is used commonly in geophysics because it gives a reliable balance between frequency and time localization [40]. Then, the wavelet coherence was used to explore the local correlation between Chl-*a* and SST, and Chl-*a* and Niño 3.4 in time and frequency space.

## Results and discussion

### Annual mean Chl-*a*

**Fig 2:**
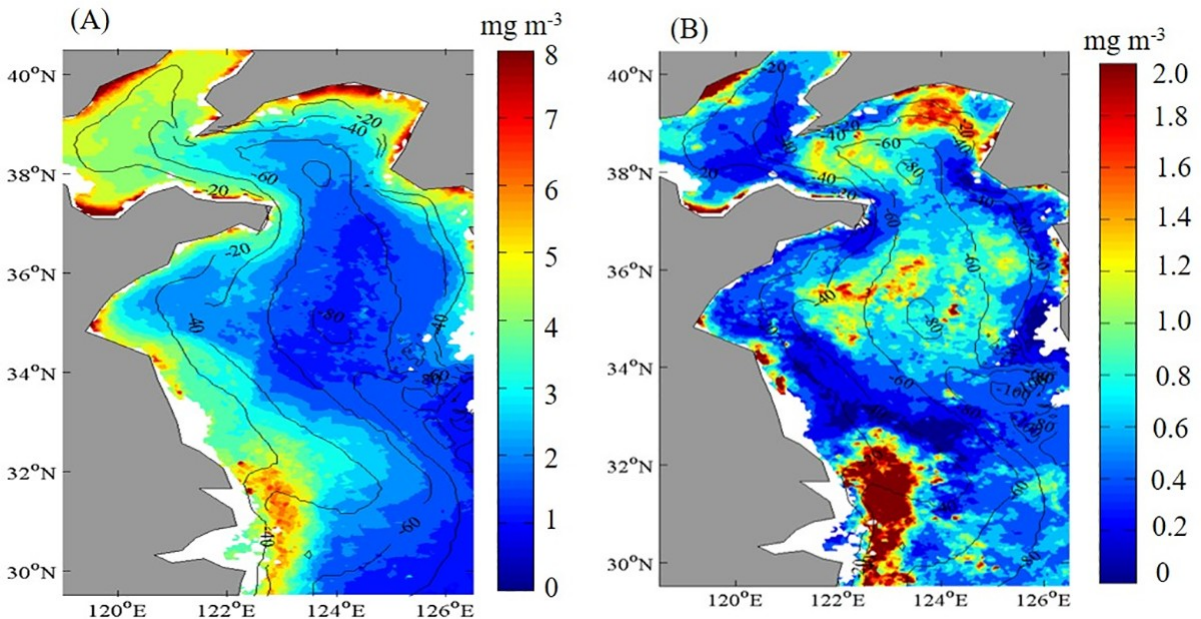
Spatial patterns of temporal means in the Yellow Sea, China. (A) Chlorophyll-*a* concentration (Chl-*a*) and (B) the Chl-*a* standard deviation map during 2003–2015.

The magnitude of Chl-*a* decreased from coastal waters to offshore regions in the Yellow Sea (Fig 2A). Normally, the highest Chl-*a* values have usually been observed in coastal waters that were adjacent to the mouth of the Yangtze River where the water depth <20 m, and the central Yellow Sea waters had one of the lowest Chl-*a* regions. Chl-*a* variability based on standard deviations (STD) of annual mean temporal values showed that the lowest STDs (~0.02 mg m^-3^) (Fig 2B) in coastal waters were observed where the highest Chl-*a* values occurred (Fig 2A). The Chl-*a* STD in the area of the YSCWM exhibited high variability, which implied that the dynamic of the YSCWM had influenced the Chl-*a* variation in the central Yellow Sea profoundly.

### Seasonal variability in Chl-a

**Fig 3:**
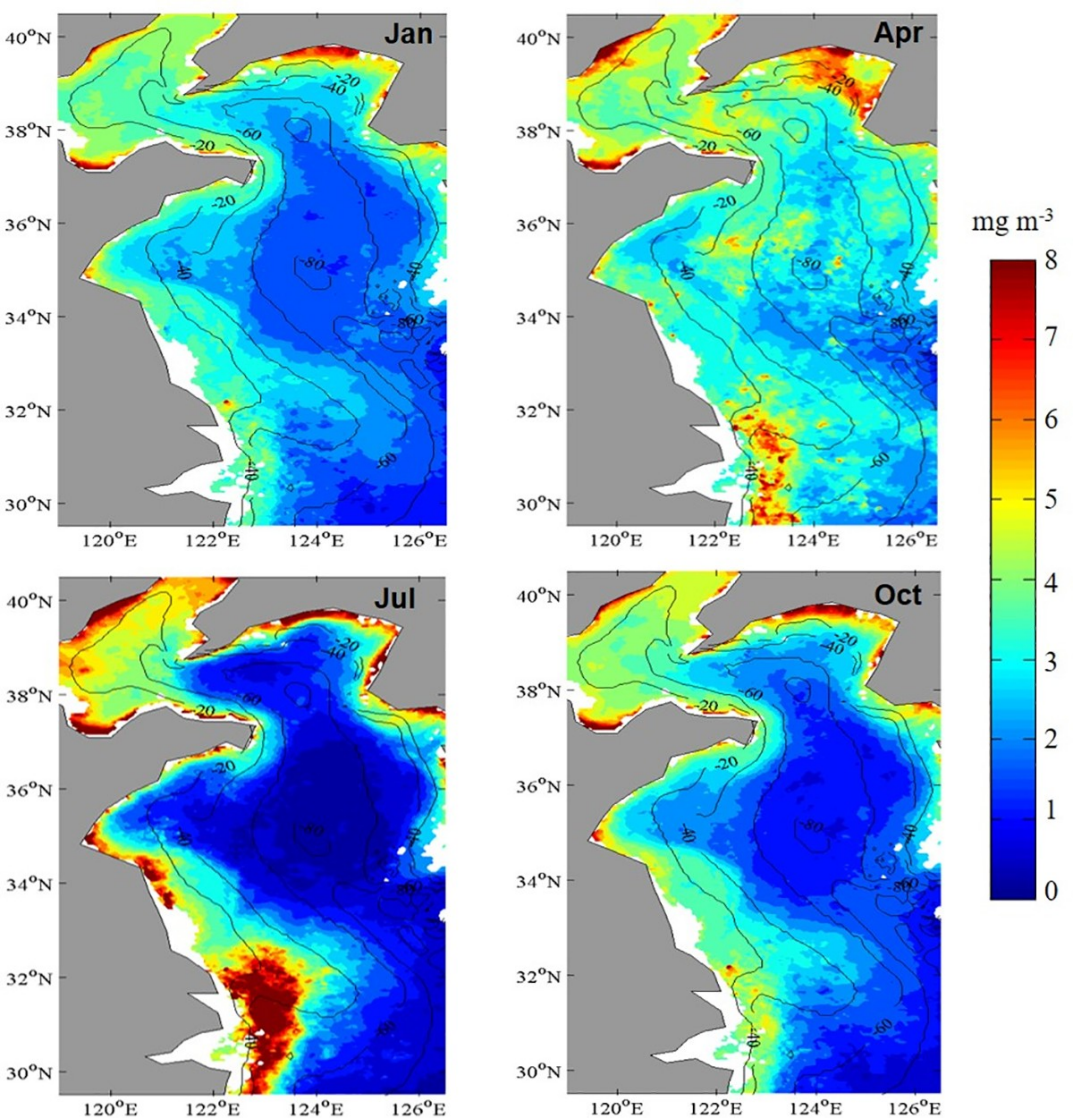
Monthly mean distributions of Chl-*a* during 2003–2015 in the Yellow Sea, China.

Although the maximum Chl-*a* appeared to be fairly consistent seasonally, the spatial extent of blooms had significant seasonal fluctuations (Fig 3). Monthly mean imagery showed that the largest spatially extent of Chl-*a*, (> 2 mg m^-3^) was in April (spring), and that the smallest extent was in July (summer) in the Yellow Sea. Chl-*a* in coastal waters (< 50-m isobaths) was relatively high in spring every year. However, some portions of phytoplankton blooms occurred only in subsurface waters, which were impossible to detect using satellite imagery [41]. Overall, the highest Chl-*a* occurred in spring in coastal regions or in regions where the seawater had been diluted greatly by freshwater, such as those close to the Yangtze River mouth, where Chl-*a* was characterized by a long-lasting summer maximum that started in April and ended in September. However, vertical mixing in the water column that was caused by the strong northeast monsoons re-suspended the sediment in this area [42], and, therefore, Chl-*a* was probably overestimated [32, 43].

**Fig 4:**
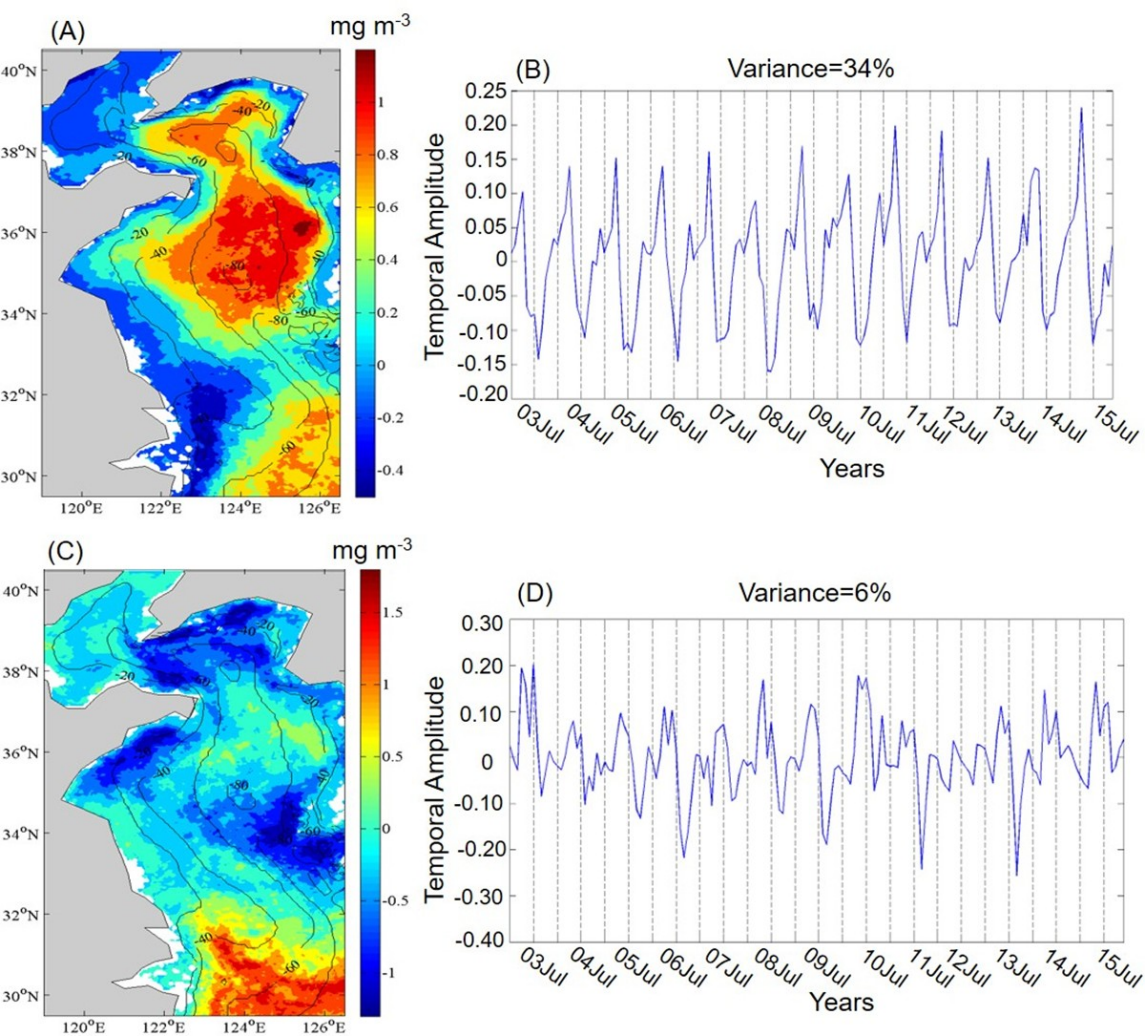
Variability in Chl-*a* during 2003–2015 in the Yellow Sea, China. (A) Spatial pattern and (B) temporal amplitude for the first EOF mode, (C) spatial pattern and (D) temporal amplitude for the second EOF mode.

The EOF was used to analyze the seasonal variability in Chl-*a*. The first and second EOF modes explained 34% and 6% of the total Chl-*a* variability in this study, respectively (Fig 4). The EOF proportions were similar to those observed in previous studies (35% and 47%) on regional and global Chl-*a* [9, 29]. The spatial pattern of the first EOF mode for Chl-*a* was not distributed uniformly throughout the entire study area (Fig 4A). One of the centers of higher Chl-*a* anomalies lay in an area at 35–37° N and 122–126° E, which was affected mainly by the YSCWM in the central waters of the Yellow Sea [18]. In that location, the strong mixing during winter brought the deeper nutrients upward in the water column, which favored phytoplankton blooms [31]. Another high Chl-*a* center was located in the southeastern region near the YSWC. Thus, we speculate that in the Yellow Sea the water masses from YSCWM and YSWC were the main factors that affected the first EOF mode of Chl-*a*. The temporal amplitude showed positive values from winter to spring (November to April) but negative values from summer to fall (June to October) (Fig 4B). The seasonal cycles could be the main reason for this phenomenon, with a high Chl-*a* occurred during winter to spring in offshore regions, and a high Chl-*a* during summer occurred in coastal areas. Also, the subsurface slope water was nutrient rich and, therefore, provided important nutrients from the subsurface to the surface [35]. The spatial pattern of the second EOF mode for Chl-*a* showed a remarkable positive signal in the southeastern Yellow Sea, but a negative signal dominated the northern Yellow Sea (Fig 4C). The second Chl-*a* mode probably represented the differences in the timing of blooms. The distribution of the second Chl-*a* EOF mode differed from that of a previous study by Liu and Wang [31], in which a remarkable positive signal was found in the central area of the Yellow Sea. The temporal amplitude of this mode exhibited a positive signal in spring (March-May) and reached maximum values in June or July. Yamaguchi et al. [32] also detected Chl-*a* maxima in the southeast region during this period.

**Fig 5:**
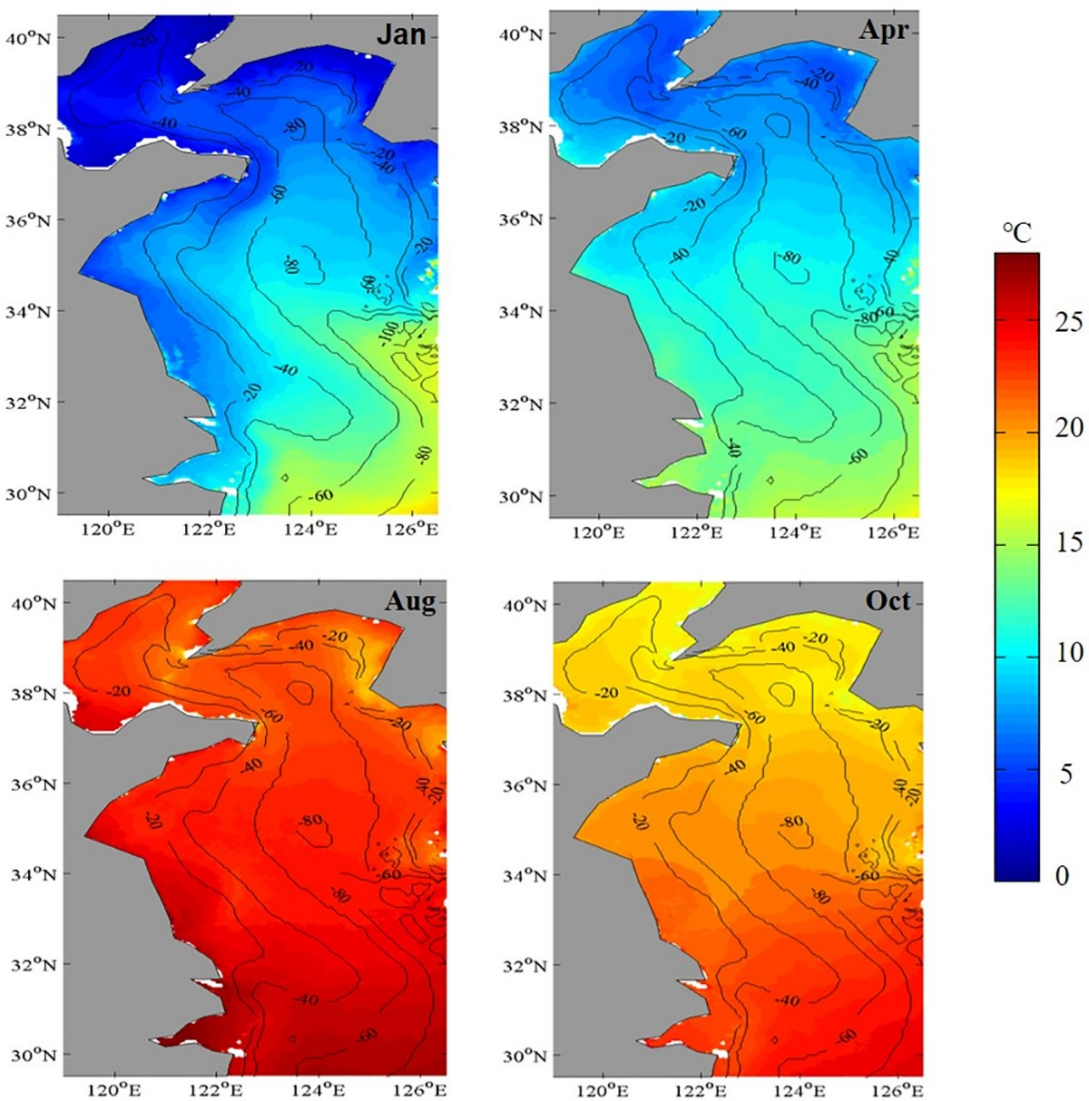
Monthly mean distributions of SST in the Yellow Sea, China during 2003–2015.

In the Yellow Sea, the SST presented a sinusoidal seasonal cycle from winter to summer, with a persistent, seasonal warming trend (Fig 5). It showed that the spatial distribution pattern of SST was opposite to that of Chl-*a* in Fig 3 (i.e., the mean Chl-*a* was higher in coastal regions than those in offshore regions, whereas the SST was lower in coastal regions than those in offshore regions). From summer to fall and winter to spring, an indistinct spatial variability was observed both for Chl-*a* and SST. This suggested that the spatial pattern of SST was contrary to that of Chl-*a* to a certain extent. The strong similarity in spatial pattern between the mean Chl-*a* and SST patterns throughout the entire Yellow Sea region was due to lower levels of phytoplankton biomass that corresponded to stronger stratification [23, 44] and the warmer surface waters in the water column [24]. A stratified water column resulted in depleted Chl-*a* in summer, and a less stratified water column resulted in increased Chl-*a* in winter [2, 23, 45]. The decreased/increased stratification was closely related to the enhanced/reduced nutrient availability [46].

### Inter-annual variability in Chl-a

**Fig 6:**
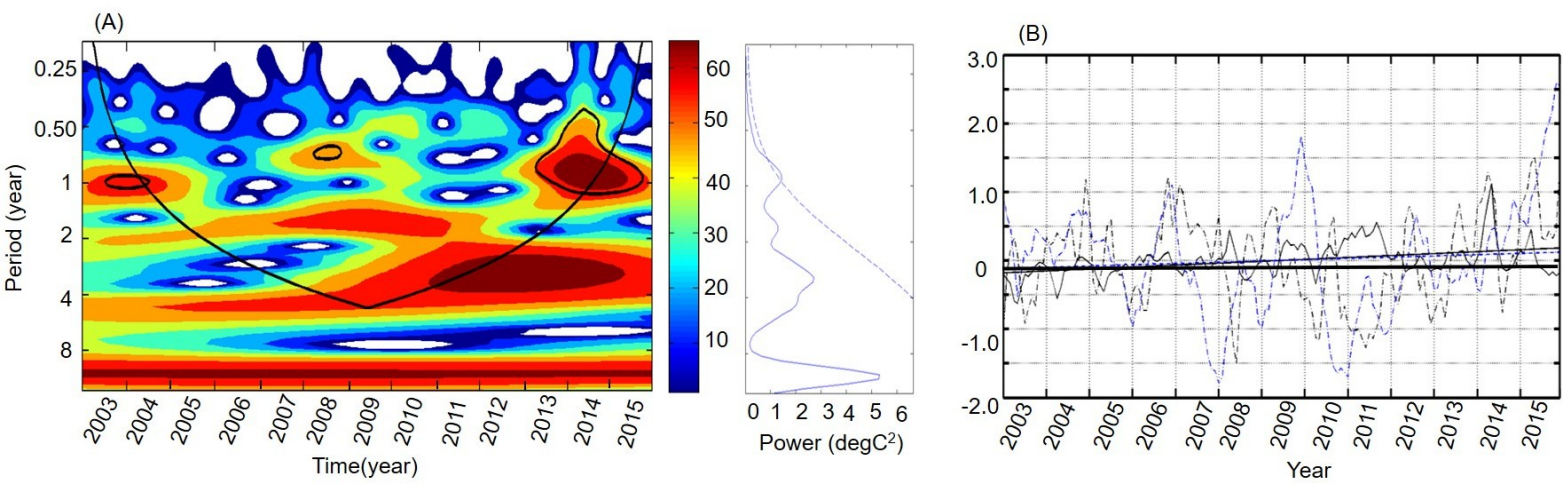
Variation in Chl-*a*, SST, and Niño 3.4 in the Yellow Sea, China. (A) Wavelets of the amplitudes for Chl-*a* after seasonal variation had been removed (the blue dotted line shows the 95% confidence level, and the solid blue line is the global power spectrum in the right panel, respectively). The warm color indicates high power in an arbitrary unit. (B) Time series of mean anomalies for Chl-*a*, SST, and Niño 3.4 (the black lines are Chl-*a* anomalies and least-square linear fits; the black dotted lines are SST anomalies and least-square linear fits; the blue lines are Niño 3.4 and least-square linear fits).

The dominant variability scales and oscillation periods of Chl-*a* were highlighted by the Morlet wavelet transform in this study. The global power spectra showed the multi-period for Chl-*a* (right panels of Fig 6A). Chl-*a* exhibited dominant and significant periods of about 1 year and insignificant periods of 3–4 years. The wavelet power spectrum showed that the power of the frequency and amplitude in Chl-*a* anomalies varied with time (left panels of Fig 6A). During 2003–2012, there was a period of variation in Chl-*a* that lasted 1.5–2 years. During 2012–2015, a significant period shift was observed at 3–4 years. Overall, during 2003–2015 Chl-*a* exhibited dominant variations at periods of 1 year and 2–4 years.

The monthly time series of the Chl-*a* anomalies, SST anomalies and Niño 3.4 are displayed in Fig 6B. The Chl-*a* and SST both exhibited increasing trends (Fig 6B), although the Chl-*a* and SST were still negatively correlated (r = -0.21, p<0.01) in the Yellow Sea, with time lags of 4 months (Chl-*a* ahead). These results agreed well with previous studies, which indicated that SST had a negative relationship with Chl-*a* [14–15, 26]. The correlation of Chl-*a* and Niño 3.4 showed a significant positive correlation (r = 0.46, p<0.01) with a lag of 3–5 months (Niño 3.4 ahead), which suggested that ENSO was probably the dominant factor that drove Chl-*a* variation on an inter-annual scale in the Yellow Sea.

**Fig 7:**
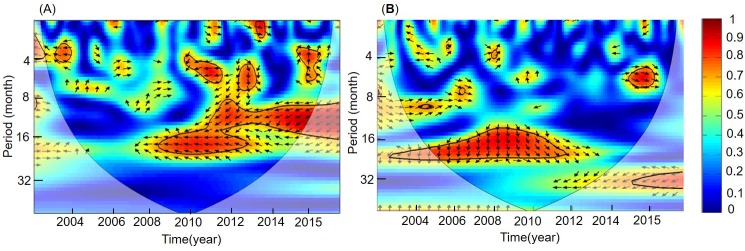
Wavelet coherency analysis of variables in the Yellow Sea, China. (A) Wavelet coherency between Chl-*a* and SST during 2003–2015 in the Yellow Sea. (B) Wavelet coherency between Chl-*a* and Niño 3.4 during 2003–2015. (The thick lines show the 95% confidence levels, and the thin lines separate the cones of influence. The color bar indicates the intensity of the correlation, and the direction of arrows show the correlation type with the right-pointing arrows being positive and left pointing arrows being negative).

To examine further the synchrony between Chl-*a* with SST and Niño 3.4, wavelet coherence analysis revealed the coherency among them (Fig 7). The wavelet squared coherence values below the confidence level indicated that there were some randomly distributed sections. The vectors indicated the phase difference between Chl-*a* with SST and Niño 3.4 at each time and period, and there was a significant negative coherency between the Chl-*a* and SST during 2008–2015, with a periodicity of 1–2 years (Fig 7A). This coherency period was consistent with the Chl-*a* and Niño 3.4 period at inter-annual scales (Fig 7B).

## Concluding remarks

The main purpose of this study was to identify the variability in Chl-*a* on both seasonal and inter-annual time scales and its cross relationship based on long-term, remotely-sensed data. In addition to the EOF, we also applied the wavelet coherency analysis to Chl-*a*, SST, and Niño 3.4 to explore the relationships in a time series for 2003–2015.

On a seasonal time-scale, spatially, Chl-*a* was highest in coastal regions in spring or in regions where the seawater had been diluted greatly by freshwater, and the SST spatial pattern was correlated highly with the distribution of water bathymetry in the Yellow Sea. Temporally, the first EOF mode for Chl-*a* exhibited stronger seasonal variability with a maximum in late winter and early spring and a minimum in summer and early fall, which was affected mainly by the water mass of YSWC and YSCWM. The second EOF mode for Chl-*a* showed differences beginning at the time of blooms. The temporal amplitude exhibited a positive signal in spring (March-May), and it reached maximum values in June or July.

On an inter-annual timescale, Chl-*a* showed dominant variations at periods of 1 year and 2–4 years during 2003–2015. There were increasing trends for Chl-*a* and SST. Furthermore, a significant negative correlation existed between Chl-*a* and SST, but there was a positive correlation between Chl-*a* and Niño 3.4. Thus, on a seasonal scale, phytoplankton variation was controlled mainly by water mass, but on an inter-annual scale, phytoplankton variation was affected by different factors (e.g., water masses, SST, ENSO) in the Yellow Sea.
